# Manufacturing of quantum-tunneling MIM nanodiodes via rapid atmospheric CVD in terahertz band

**DOI:** 10.1038/s41598-023-47775-5

**Published:** 2023-11-25

**Authors:** Dogu Ozyigit, Farman Ullah, Ahmet Gulsaran, Bersu Bastug Azer, Ahmed Shahin, Kevin Musselman, Michal Bajcsy, Mustafa Yavuz

**Affiliations:** 1 Mechanical and Mechatronics Engineering Department, University of Waterloo, Waterloo, ON N2L 3G1 Canada; 2 Waterloo Institute for Nanotechnology (WIN), University of Waterloo, Waterloo, ON N2L 3G1 Canada; 3 Department of Electrical and Computer Engineering, University of Waterloo, Waterloo, ON N2L 3G1 Canada; 4 Institute for Quantum Computing, University of Waterloo, Waterloo, ON N2L 3G1 Canada

**Keywords:** Engineering, Nanoscience and technology, Materials science

## Abstract

Quantum-tunneling metal–insulator-metal (MIM) diodes have emerged as a significant area of study in the field of materials science and electronics. Our previous work demonstrated the successful fabrication of these diodes using atmospheric pressure chemical vapor deposition (AP-CVD), a scalable method that surpasses traditional vacuum-based methods and allows for the fabrication of high-quality Al_2_O_3_ films with few pinholes. Here, we show that despite their extremely small size 0.002 µm^2^, the MIM nanodiodes demonstrate low resistance at zero bias. Moreover, we have observed a significant enhancement in resistance by six orders of magnitude compared to our prior work, Additionally, we have achieved a high responsivity of 9 AW^−1^, along with a theoretical terahertz cut-off frequency of 0.36 THz. Our approach provides an efficient alternative to cleanroom fabrication, opening up new opportunities for manufacturing terahertz-Band devices. The results of our study highlight the practicality and potential of our method in advancing nanoelectronics. This lays the foundation for the development of advanced quantum devices that operate at terahertz frequencies, with potential applications in telecommunications, medical imaging, and security systems.

## Introduction

The projected increase in wireless data traffic for the upcoming decade is encouraging both academia and industry to think beyond current wireless technologies and consider the potential of sixth-generation (6G) wireless networks. Among a range of advanced and promising solutions, terahertz (THz) communications, which operate within the ultra-wide THz band from 0.1 to 10 THz, are seen as highly promising for the 6G era and beyond. This is due to their unique capability of supporting terabit-per-second transmission in emerging applications^1^. This advancement has consequently led to the development of affordable, scalable methods for manufacturing THz devices. MIM diodes are one of the promising candidates for THz applications^2^. These unique diodes, which feature a thin insulating layer sandwiched between two metal contacts, employ a quantum-tunnelling electron transport mechanism, making them well-suited for high-frequency signal rectification. Their primary applications include solar rectennas^3^, infrared detectors^1,4^, and wireless power transmission systems^5^.

MIM diodes play a crucial role in the rapidly advancing field of THz technology, demanding a more in-depth analysis of their fabrication methods^6^. The performance of these diodes heavily relies on the quality of the insulating layer. Traditional oxidation techniques, including native, thermal, plasma, and anodic oxidation, are used to grow an oxide layer over the base metal electrodes such as Ni, Ti, and Al.

However, achieving consistent oxide quality and thickness using these methods poses challenges due to varying conditions of humidity and partial pressure of oxygen in the air. In contrast, a more promising approach known as in situ oxidation offers controlled results and lower defect density^7^. The limitations of oxidation methods lie in their ability to produce only derivative oxides of the base metal. To overcome this constraint, deposition techniques such as sputtering^8^, electron beam evaporation^9^, pulsed laser deposition^10^, and Atomic Layer Deposition (ALD)^11^ have been developed. Among these, ALD stands out as it produces high-quality oxides with low defect density and excellent conformality, making it suitable for insulator deposition in MIM fabrication. However, their usage is mostly limited by slow deposition rates and the requirement for a vacuum environment. These limitations hinder the scalability of MIM diode production, a critical aspect of their integration into the growing THz field. In response to the urgent need for scalable deposition techniques for nanoscale films, which are essential for the development of integrated quantum devices, we employed the Atmospheric Pressure Spatial Atomic Layer Deposition (AP-SALD) method. This method enables the fabrication of Al_2_O_3_ insulating films for MIM nanodiodes. AP-SALD offers several advantages, including speed, scalability, operation at atmospheric pressure in an open-air environment, and precise control of film thickness at the atomic scale. Moreover, it can produce conformal and high-quality films at moderate temperatures, ranging from 80 to 200 °C^12^. By adjusting the gas phase environment to incorporate precursors, the same AP-SALD system can also facilitate Chemical Vapor Deposition (CVD) under AP-CVD conditions^13^. AP-CVD is particularly advantageous for device fabrication since it achieves higher deposition rates while maintaining smooth, defect-free, and conformal films with thicknesses corresponding to the number of deposition cycles^14^. The main objective of our research is to develop diodes that operate effectively in the THz frequency range. We will compare the performance of MIM diodes fabricated through AP-CVD with those created using vacuum-based Plasma Enhanced Atomic Layer Deposition (PEALD). The implementation of AP-SALD under AP-CVD conditions could represent a significant advancement in the consistent production of high-quality MIM diodes for the THz field. This, in turn, could potentially reduce manufacturing costs, enhance production speed, and enable greater scalability. Therefore, our research could accelerate the development of quantum devices that work in the Terahertz gap.

## Results and discussion

**Figure 1:**
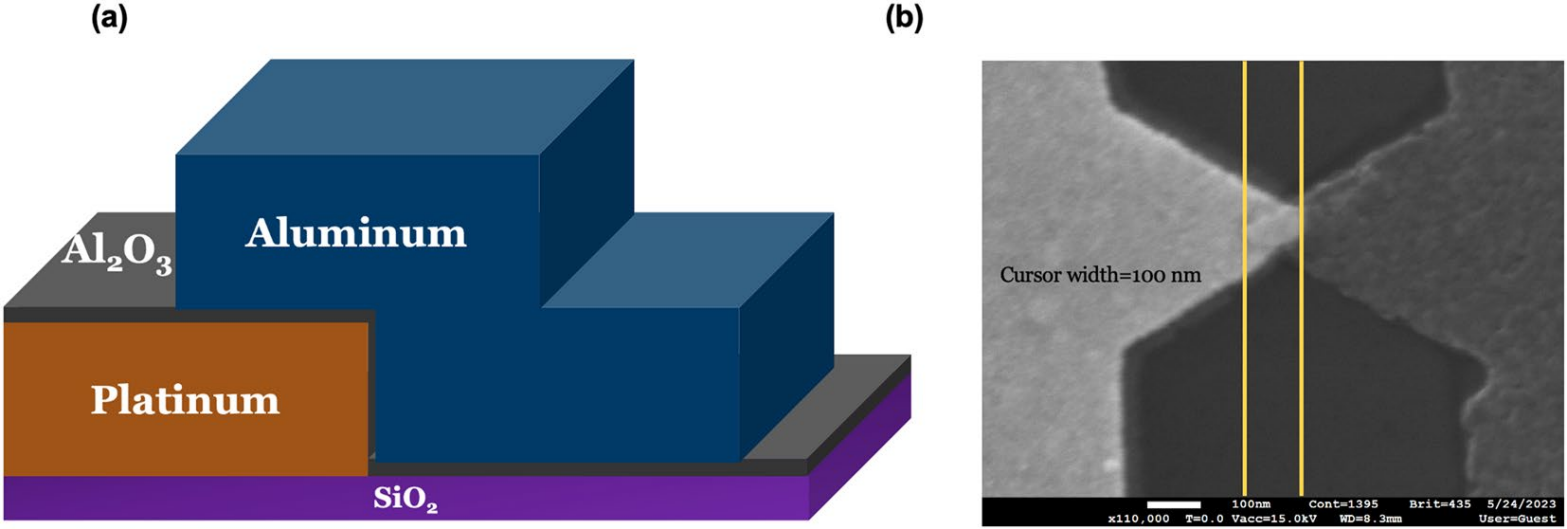
(**a**) Schematic illustration of cross-sectional view of the Pt–Al_2_O_3_–Al MIM diode device architecture after deposition of the top Al electrode and patterning steps. and (**b**) scanning electron microscopy (SEM) image of fabricated MIM diode. The contact area is 0.002 µm^2^.

Figure 1a shows a diagram of cross-sectional view Pt–Al_2_O_3_–Al MIM diodes that we fabricated. The wafers used were p-doped silicon wafers with a thermally grown 285 nm oxide layer. Next, we patterned the first metal electrodes using e-beam lithography. Pt was deposited using an e-beam evaporation process. Following this step, we deposited an Al_2_O_3_ layer on top of the Pt. In our experiments, this layer had different thicknesses ranging from 3 to 9 nm. The Al_2_O_3_ layer was deposited at 150 °C using AP-CVD and PEALD systems. The details of these deposition conditions are elaborated in the “Experimental section” of our paper.

**Figure 2:**
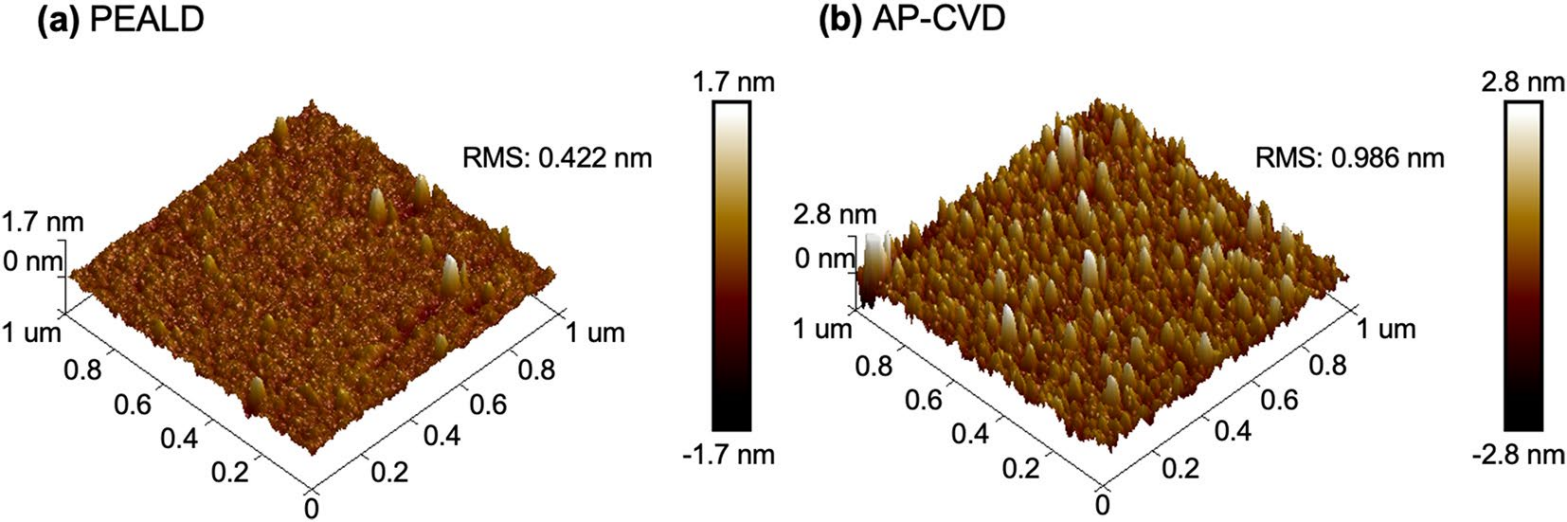
3D Atomic force microscope images of (**a**) 9 nm Al_2_O_3_ by PEALD, (**b**) 9 nm Al_2_O_3_ by AP-CVD.

The consistency of the insulating layer in MIM diodes is crucial for their overall performance and stability. Variations in the insulator’s thickness due to the presence of pinholes can lead to inconsistencies in the device, causing alterations in the current density and subsequently the tunneling probability. These deviations arise due to the exponential relationship between the current and the electric field in these systems^15^. The presence of unintended defects such as pinholes or traps within the oxide film can lead to conduction mechanisms that are often regarded as detrimental. These issues originate from the metal/oxide interface and can cause substantial external damage to the device, as well as internal damage from defects, reducing its effectiveness and reliability. Therefore, achieving a consistently thin and defect-free insulating layer is not just desirable but crucial for the efficient and reliable operation of MIM diodes. The surface roughness of aluminum oxide (Al_2_O_3_) thin films, which were deposited using PEALD and AP-CVD, was evaluated in this study (see Fig. 2). The roughness of the metal–insulator interface in MIM diodes plays a vital role in determining the tunnelling current and overall performance of the device. A smoother surface of the insulator can improve the breakdown voltage, resulting in a more durable device. On the other hand, increased roughness can lead to higher leakage current, which is undesirable in various MIM diode applications. Additionally, the uniformity of the electric field is critical, non-uniform thickness can cause non-uniform capacitance, which might potentially impact the efficiency of the device in high-frequency applications^6,16^. Despite the less controlled conditions of an open-air setting, the AP-CVD process produced a thin film with a surface roughness of only 0.986 nm, demonstrating its efficacy in maintaining a low level of roughness. Conversely, the PEALD technique exhibited superior performance, achieving a remarkably low root mean square (RMS) roughness of 0.422 nm, indicating its dominance in achieving highly smooth surfaces.

In our study, we used Aluminum (Al) as the secondary metal layer, which was deposited on the insulator through the e-beam evaporation process. It is also patterned by E-beam lithography, and it resulted in a device area of roughly 0.002 µm^2^. The contact area was determined through SEM, as illustrated in Fig. 1b. We chose Platinum–Aluminum (Pt–Al) as the electrode pair for two main reasons. Firstly, their significant work function difference is one of the highest reported in current literature at 1.37 eV^17^. This difference is crucial for obtaining superior performance metrics. Secondly, the combination of Al and the noble metal Pt as electrode materials significantly reduces the chance of forming unwanted oxides at the interfaces, except for aluminum oxide. For devices that utilized PEALD to incorporate Al_2_O_3_ films, we employed a fabrication process similar to the AP-CVD method. Specifically, we selected trimethylaluminum (TMA) as the metal precursor and applied an oxygen (O_2_) plasma during the deposition process. We performed 27, 54, and 81 cycles to achieve film thicknesses of 3, 6, and 9 nm, respectively. We used ellipsometry to verify these measurements. It is worth noting that Atmospheric Pressure Chemical Vapor Deposition (AP-CVD) was more time efficient than PEALD. While the PEALD took 210 s to create a 6 nm film, AP-CVD achieved a similar thickness for Al_2_O_3_ in just 15 s.

**Figure 3:**
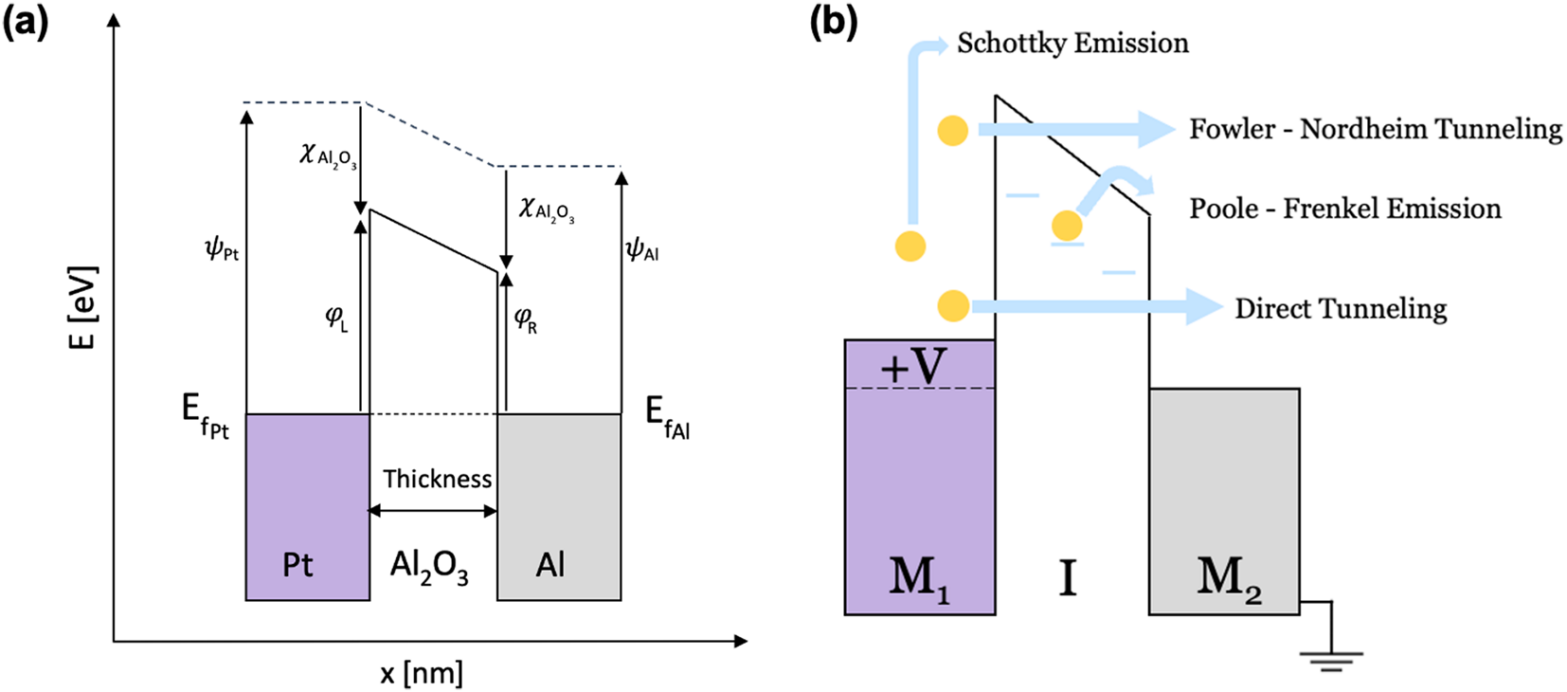
(**a**) Energy diagram of the Pt–Al_2_O_3_–Al MIM diode. (**b**) Representation of Schottky Emission, Fowler–Nordheim Tunneling, Poole–Frenkel Emission, Direct tunneling conduction mechanisms in asymmetric positive biased MIM diode.

The energy band diagram for the Pt–Al_2_O_3_–Al MIM diode, including potential barriers at the interfaces, is shown in Fig. 3a. The barrier height (*φ*) is calculated as the difference between the metal’s work function (*ψ*) and the insulator’s electron affinity (*χ*). The Al_2_O_3_ produced by PEALD has an electron affinity of 1.90 ± 0.2 eV, as reported in previous studies^18^, while the work functions of Pt and Al are approximately 5.69 eV^19^ and 4.28 eV^20^, respectively. As a result, we expect a left barrier height (*φ* L) of approximately 3.79 eV at the Pt–Al_2_O_3_ interface, and a right barrier height (*φ* R) of about 2.38 eV at the Al–Al_2_O_3_ interface. The thickness of the insulator affects the width of the barrier. We explored MIM diodes based on two different processes—AP-CVD and PEALD. These were studied over a voltage span in a range from − 1 V to + 1 V. Conduction mechanisms in an asymmetric positive biased MIM diode are shown in Fig. 3b. In the case of Poole–Frenkel conduction, the movement of electrons through the insulator via trapped states is responsible for conduction. In contrast, Schottky emission is a thermal activation process where conduction occurs as electrons move across the metal–insulator barrier. On the other hand, quantum electron tunnelling can occur across the thin insulator through two distinct mechanisms: direct tunnelling and Fowler–Nordheim tunnelling (FNT). Direct tunneling involves electrons crossing the entire thickness of the barrier, while Fowler–Nordheim tunneling occurs when electrons tunnel across a shorter distance in the triangular region of the barrier, caused by the difference in metal work functions.

Developing MIM diodes that are appropriate for high-frequency applications is a considerable obstacle. The difficulty arises from the need to achieve both low resistance and high responsivity concurrently. The importance of low resistance lies in its contribution to a smaller Resistance–Capacitance (RC) time constant. A decreased RC time constant allows for a higher cut-off frequency, which is a desirable characteristic in high-frequency applications^21^. In order to create an effective rectenna, it is crucial to have a strong connection between the MIM diode and the antenna. For optimal power transfer, the impedance of the antenna needs to match the impedance of the diode. Additionally, efficient rectification requires a high diode responsivity, which quantifies the rectified DC voltage or current produced in response to input power. Responsivity is a metric that gauges the MIM diode’s capacity to generate direct current (DC) when exposed to alternating current (AC) electrical power. This parameter is determined by dividing the second derivative of the I–V curve by twice the first derivative at a given bias voltage^22^.

1 $$ Responsivity = \frac{1}{2}\left( {{{\frac{{d^{2} I}}{{dV^{2} }}} \mathord{\left/ {\vphantom {{\frac{{d^{2} I}}{{dV^{2} }}} {\frac{dI}{{dV}}}}} \right. \kern-0pt} {\frac{dI}{{dV}}}}} \right) $$

The responsivity equation is shown in Eq. (1). *I* is the current in amperes (A), *V* is the voltage in volts (V). Responsivity is influenced by multiple factors including the choice of metal and insulator, the thickness of the insulator, and the barrier heights between the metals and the insulator.

Table 1 presents a comparison of diode performance using different fabrication methods for Al_2_O_3_ films in various studies: AP-CVD and PEALD. The parameters examined include maximum responsivity, near zero-bias responsivity, and near zero-bias resistance. In the current study, the diode produced using the AP-CVD process with a 6 nm Al_2_O_3_ layer showed significantly reduced resistance, measuring at 25 × 10^6^ Ω. This resistance level is notably lower than what was observed in previous research findings^17^. This improvement can be attributed to the replacement of reactive ion etching (RIE) with wet etching for removing the oxide layer on top of the electrode. The motivation behind shifting from RIE to wet etching was to decrease contact resistance. In RIE, the surface is bombarded by ions in a non-selective manner, which could increase the roughness of the metal layers and consequently raise the contact resistance in previous studies. On the other hand, wet etching is a gentler and more uniform process that preserves the integrity of the underlying material. As a result, it produces a smoother surface, and research conducted by Hisakado^23^ has shown a correlation between smoother surfaces and lower contact resistance. Furthermore, wet etching facilitates a cleaner interface between the aluminum and the contact electrode, reducing the potential risks associated with a rough surface. To enhance these advantages, we deposited a gold layer immediately after the oxide wet etch to prevent further oxidation of the aluminum layer. Overall, both our experimental results and existing findings indicate that transitioning from RIE to wet etching can lead to the creation of smoother and more stable interfaces, resulting in reduced contact resistance.

**Table 1:** Comparison of near zero-bias resistance, near zero-bias responsivity, and maximum responsivity values for diodes with a 6 nm Al_2_O_3_ layer deposited using PEALD and AP-CVD methods.

	Near zero-bias resistance (Ω)	Near zero-bias responsivity (AW^*−*1^)	Maximum responsivity (0–1 V) (AW^*−*1^)
6 nm PEALD Diodes (Current Study)	40 × 10^6^	0.4	6.1
6 nm AP-CVD Diodes (Current Study)	25 × 10^6^	0.2	5.02
6 nm PEALD Diodes (Prior Study^17^)	7 × 10^12^	− 1	3.5
6 nm AP-CVD Diodes (Prior Study^17^)	5 × 10^12^	6.5	6.5

This comparison encompasses data from the current study as well as a previous study.

**Figure 4:**
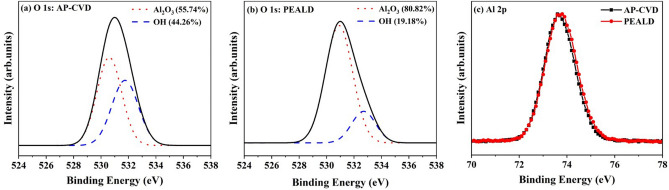
XPS spectra of Al_2_O_3_ films (**a**) O 1s: AP-CVD, (**b**) O 1 s: PEALD, and (**c**) Al 2p.

The AP-CVD diodes fabricated with a 6 nm Al_2_O_3_ layer demonstrate lower resistance compared to PEALD diodes, with the resistance measured at approximately 40 × 10^6^ Ω. This observed difference can be attributed to the presence of defects in the AP-CVD diodes. The X-ray photoelectron spectroscopy (XPS) results depicted in Fig. 4 reveal, AP-CVD diodes exhibit a higher concentration of OH^–^ ions.

**Figure 5:**
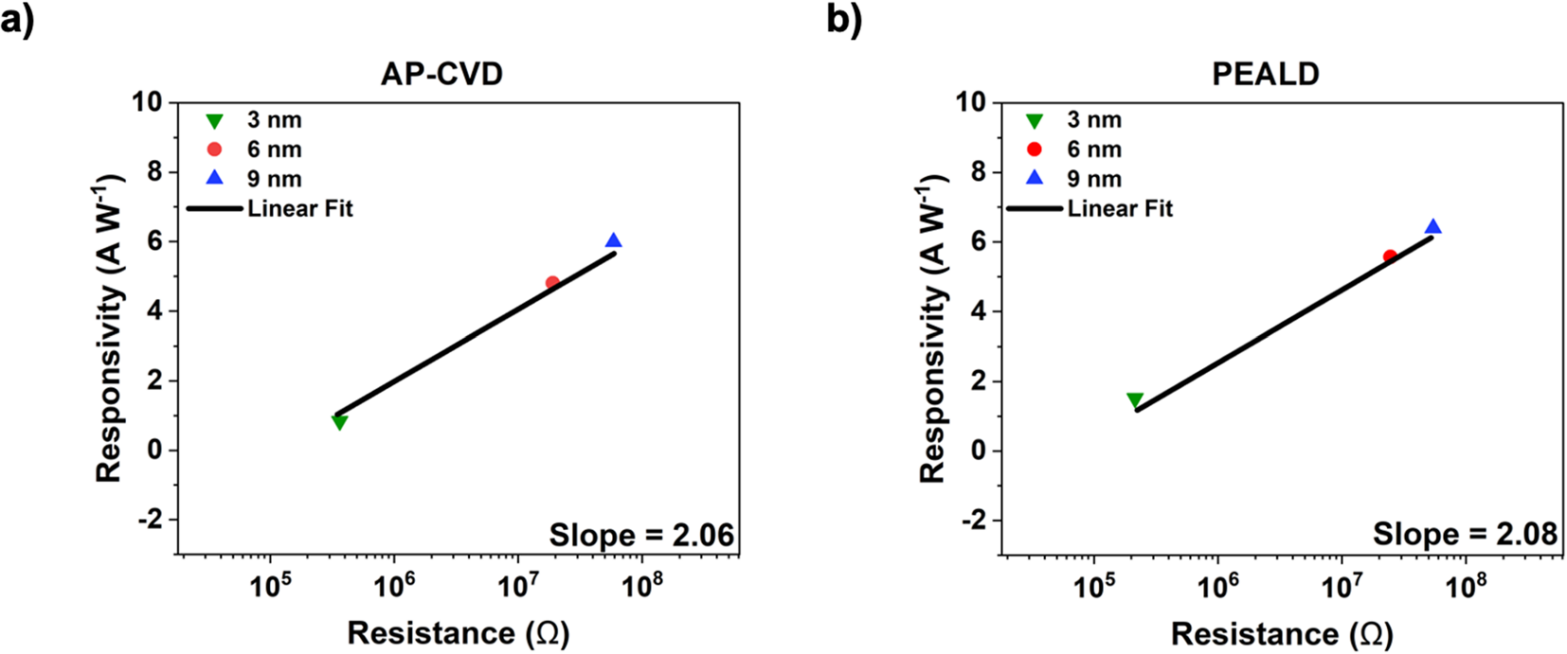
Graph shows the relationship between responsivity and resistance for (**a**) AP-CVD and (**b**) PEALD diodes across varying insulator thicknesses (3 nm, 6 nm, and 9 nm) at 0.60 V bias. The graph’s slope denotes the Responsivity/Resistance ratio.

The presence of these ions facilitates enhanced Poole–Frenkel (PF) conduction, which in turn leads to a decrease in resistance. This observation aligns with findings from previously reported studies^17,24^. Moreover, this work on PEALD shows a doubled maximum responsivity value, which can be attributed to improved contact between the probe station and electrodes, while the maximum responsivity value for AP-CVD remains unchanged. Figure 5 illustrates the relationship between Responsivity and Resistance for the diodes that were produced. By employing a thicker insulator, the occurrence of electron tunnelling is minimized, resulting in a lower on-state current. However, this also enhances the responsivity of the diode. The slope of the graph represents the Responsivity/Resistance ratio, where a higher value indicates improved rectification performance at high frequencies. Achieving high rectification efficiency requires a high responsivity, as well as low resistance for optimal antenna coupling and a low RC constant. Figure 5 indicates a similar slope for AP-CVD (2.06) and PEALD (2.08). Based on the slopes, it can be inferred that diodes created using AP-CVD could potentially demonstrate compatible performance at high frequencies when compared to diodes produced using PEALD. Nevertheless, when considering variables such as production cost and processing speed, AP-CVD emerges as a more advantageous option compared to PEALD. Also, the AP-CVD diode’s performance has been observed to be competitive with previously reported THz diodes that utilized a single layer of Al_2_O_3_^25^ and PEALD fabricated diodes. This comparison is based on the parameters of responsivity and resistance. The exceptional performance characteristics exhibited by AP-CVD diodes can be attributed to various factors, such as the conduction mechanisms involved, the barrier heights between the metals and insulator, surface roughness, and the presence of defects in the insulator^26^. The anticipated conduction processes in MIM diodes encompass three significant mechanisms: Poole–Frenkel (PF), Schottky emission (SE), and quantum electron tunnelling.

**Figure 6:**
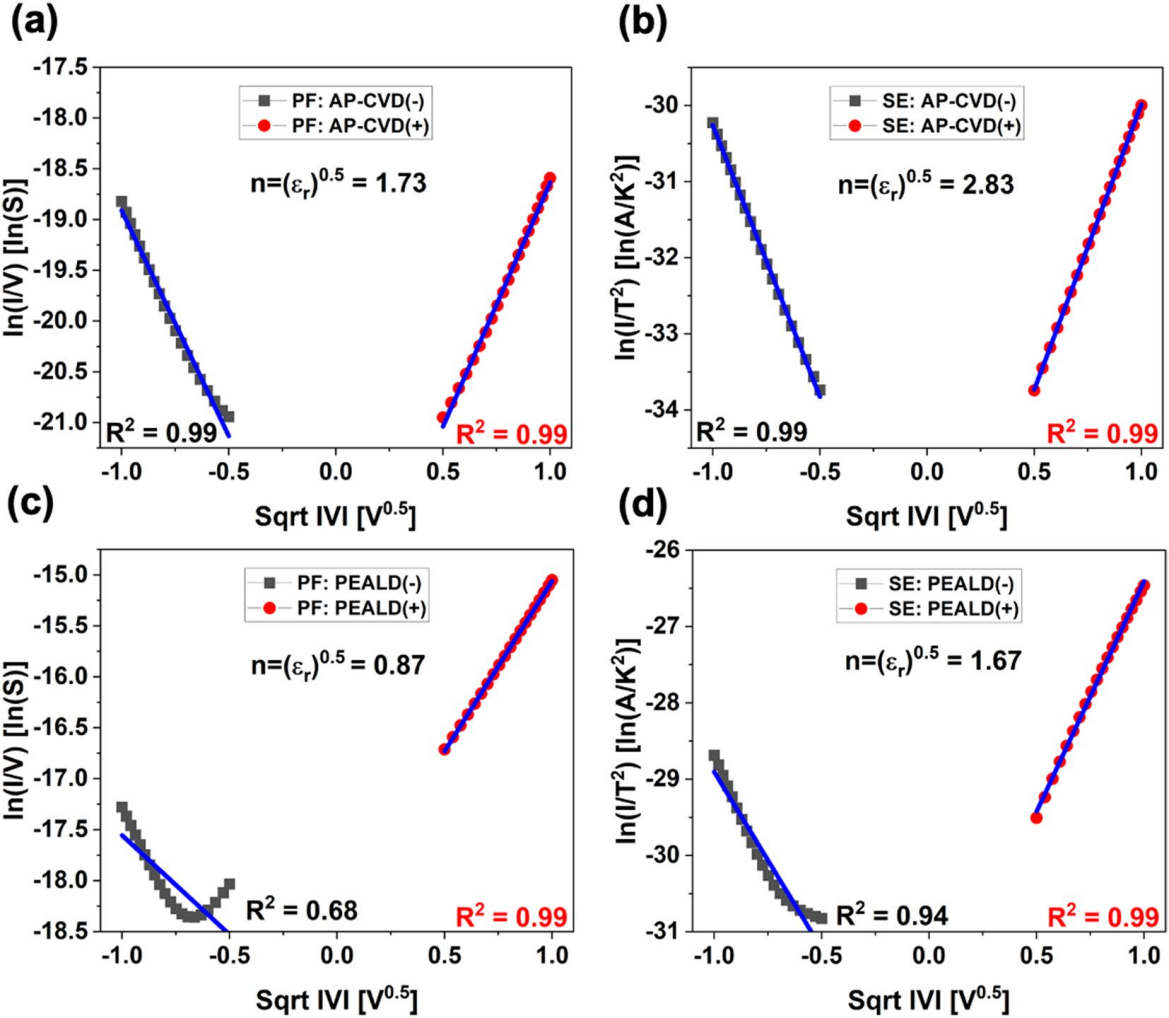
Diodes made with AP-CVD and PEALD exhibit two main conduction mechanisms: (**a**, **c**) Poole–Frenkel and (**b**, **d**) Schottky emission. The conduction mode is indicated by black squares for negative polarity and red circles for positive, pointing to a dominant mechanism. Correlations with an R^2^ value above 0.99 confirm this dominance and corresponding refractive indices are provided.

We determined the relative permittivity (*ε*_*r*_) of the AP-CVD and PEALD diodes using equations derived from Poole–Frenkel, Schottky Emission, and Fowler–Nordheim that are shown in the supporting document. Our results are consistent with our recent studies^17^. We observed that at lower voltage biases, Poole–Frenkel conduction was the primary mechanism in AP-CVD diodes, while Schottky emission was the dominant mechanism in PEALD diodes under similar conditions. At higher voltage biases, regardless of the Al_2_O_3_ deposition method used, Fowler–Nordheim tunnelling emerged as a significant conduction mechanism. Figure 6 display the I–V data for the AP-CVD diode, with (a) Poole–Frenkel and (b) Schottky emission fits at low voltage biases (0.09–1 V). The Poole–Frenkel fits in Fig. 6a (R^2^ = 0.99 and R^2^ = 0.99) and Schottky emission fits in Fig. 6b (R^2^ = 0.99 and R^2^ = 0.99) show similar results. However, the extracted dielectric constants differ between these fits. The *ε*_*r*_ obtained from the Poole–Frenkel fits in Fig. 6a is 3.06, while the Schottky emission fits in Fig. 6b indicate *ε*_*r*_ = 8. The dynamic dielectric constant, *ε*_*r*_ = *n*^2^, which was measured by ellipsometry, ranges from 1.47 to 1.74 for AP-CVD Al_2_O_3_ and 1.60–1.91 for PEALD Al_2_O_3_ (Figure S2). The value *ε*_*r*_ = 3.06 from the Poole–Frenkel fitting corresponds to a refractive index *n* = (*ε*_r_)^0.5^ = 1*.*73, which aligns well with the measured range of 1.47–1.74 by ellipsometry. These findings suggest that Poole–Frenkel conduction is the dominant mechanism in AP-CVD diodes at low voltage biases. Figure 6c and Fig. 6d illustrate the I–V data for the PEALD diode, fitted with (c) Poole–Frenkel and (d) Schottky emission relations at low voltage biases. For PEALD diodes, the Schottky emission plots in Fig. 6d yield better fits (R^2^ = 0.94 and R^2^ = 0.99) compared to the Poole–Frenkel plots in Fig. 6c (R^2^ = 0.68 and R^2^ = 0.99). Furthermore, the refractive index n = 1.67 obtained from the Schottky emission plots matches the range of 1.60–1.91 measured by ellipsometry. This indicates that Schottky emission is the dominant conduction mechanism in PEALD diodes at low voltages.

We determined the cut-off frequencies by employing Eq. (2) and obtained *ε*_*r*_ values via ellipsometry measurements. The resistance *R*, denoted in ohms, for the 3, 6 and 9 nm thick insulator diodes can be found in Fig. 5. The capacitance *C* is in farads, *ε*_0_ is the permittivity of free space, *ε*_*r*_ is the relative permittivity, *A* represents the area in square meters, and *d* denotes the insulator thickness in meters. Despite the presence of resistances on the order of MΩ, the diminutive surface area of the diodes—with the smallest reaching 0.002 µm^2^—suggests that the fabricated diodes have the potential to operate within the Terahertz Gap, which spans from 0.1 to 10 THz^27^. Cleanrooms have traditionally been considered essential for producing high-quality insulator films. However, our findings challenge this perspective, suggesting that cleanroom conditions are not invariably necessary. Specifically, diodes with a size of 3 nm fabricated through PEALD and AP-CVD processes demonstrate potential cut-off frequencies of 0.36 THz and 0.19 THz, respectively. This data supports the notion that quantum mechanical devices can be successfully produced outside stringent cleanroom facilities. Notably, while reducing the diode area may further improve the cut-off frequency, there are limitations. Scaling below 0*.*002 µm^2^ is hindered by the vertical thickness of the MIM nanodiodes. A promising strategy for boosting device performance could encompass the utilization of materials with a reduced refractive index or minimizing the resistance.

2 $$f_{cut} = \frac{1}{2\pi RC} = \frac{d}{{2\pi R\varepsilon_{0} \varepsilon_{r} A}}$$

The unique electrical properties of AP-CVD and PEALD diodes under low voltage conditions can be understood by applying XPS on Al_2_O_3_ films produced by these two methods. This analysis technique sheds light on the chemical composition and bonding structure of the material. By examining the O 1s peaks through a Gaussian fit, as shown in Fig. 4a and b, distinct peaks at 531 eV and 532.3 eV were detected. These values represent O^–2^ in Al_2_O_3_ and hydroxyl OH^*−*^ groups, respectively, as noted in reference^28^. To determine the fraction of the O 1s signal assigned to these components in the Al_2_O_3_ films produced by AP-CVD and PEALD, the areas under the associated XPS peaks were computed. As expected, the AP-CVD Al_2_O_3_ film, depicted in Fig. 4a, presented a greater concentration of OH^*−*^ groups. This is attributable to the employment of H_2_O as the oxygen precursor in the AP-CVD process, as confirmed by reference^28^. It is crucial to realize that the XPS data only cover the surface of the material, and the low-temperature thermal deposition process involving H_2_O tends to leave a substantial amount of OH^*−*^ on the film surface, as outlined in references^29^. This increased presence of OH^*−*^ could be a result of incomplete precursor reactions and the significant energy required for water to evaporate at lower temperatures, as pointed out in reference^29^. These hydroxyl groups are expected to function as electron traps in the films, encouraging Poole–Frenkel conduction at low voltages, which is in line with the dominant Poole–Frenkel conduction observed in the AP-CVD diodes in Fig. 6a, as explained in reference^30^. Contrastingly, the O 1 s peak corresponding to the Al_2_O_3_ component in the PEALD film, portrayed in Fig. 4b, is considerably higher. This can be credited to the high reactivity related to the PEALD process, which uses O^−2^ plasma and results in denser films, as previously mentioned in reference^31^, can be seen in AFM figures in Fig. 2. This conclusion is in agreement with the higher percentage of Al noticed in the PEALD film, as stated in Table S1 in the Supplementary Information, and the elevated refractive index recorded in Fig. S2 of the Supplementary Information. Furthermore, the Al 2p spectra of both the AP-CVD and PEALD Al_2_O_3_ films in Fig. 4c feature a peak at 74 eV, a value associated with Al_2_O_3_, as described in reference^32^.

## Conclusions

We have successfully developed new Pt-Al_2_O_3_-Al nanodiodes. While these nanodiodes share the same layer structure as our prior work, their size has been significantly reduced, approximately by a factor of 50,000. This small design offers potential for operations within the THz gap. These nanodiodes utilize a thin Al_2_O_3_ insulating film, which is deposited in an open-air environment using a rapid and scalable method. To achieve this, we utilized an Atomic Layer Deposition System (AP-SALD) under Chemical Vapor Deposition (CVD) conditions. This approach allowed us to create MIM diodes with high-quality insulator films without any pinholes in a shorter time compared to the conventional PEALD method.

In our investigation of MIM diodes produced using AP-CVD, we discovered that Poole–Frenkel emission was the dominant conduction mechanism at lower bias voltages. This phenomenon is consistent with the higher roughness observed on the surfaces of AP-CVD films compared to those deposited using PEALD. Moreover, it is also attributed to a higher concentration of hydroxyl groups in the films, indicating that the non-uniformity in the insulator layer results in the formation of trapped states. In contrast, MIM diodes utilizing aluminum oxide (Al_2_O_3_) deposited via PEALD exhibited remarkable uniformity and predominantly displayed Schottky emission at low bias voltages. These findings highlight the significant influence of the fabrication technique on the uniformity of the insulator layer, subsequently affecting the conduction mechanisms and overall performance of the devices. Importantly, the differences in insulator uniformity resulting from the distinct deposition methods led to variations in electrical behaviours and different figures of merit.

Furthermore, we conducted calculations to determine the theoretical cut-off frequencies for the fabricated diodes. Our results indicate that diodes created using both AP-CVD and PEALD methods can operate in the THz range. Additionally, there are opportunities for further improvements, such as reducing resistance and exploring alternative materials with different refractive indices.

While our primary focus was on a simple MIM structure, future research can expand this approach to include multi-insulator diodes. These diodes could address the challenge of high diode resistance while enhancing performance by reducing the effective tunnelling distance^11,33^. Another potential approach to enhance diode performance is enabling resonant tunnelling of electrons^21^. Recent studies have also demonstrated that a defect-engineering approach can enhance the figures of merit without causing an undesirable increase in diode resistance^24^. This scalable technique has broader applications, including resistive switching memories^34^ and metal–insulator-semiconductor diodes^35,36^. Despite the continued reliance on vacuum and lithography techniques for metal electrode deposition and device patterning, our method of depositing the insulator layer represents a significant step toward the cost-effective and scalable manufacturing of THz devices.

## Experimental section

### Materials and deposition

A 100 nm thick bottom Platinum (Pt) electrode was deposited on p-type Si substrates with a 285 nm thermal SiO_2_ layer using an Angstrom E-beam evaporation system. The base pressure was set to 4 × 10^*−*6^ torr and the deposition rate was 3 A˚ s^−1^. The Pt electrodes were patterned using E-Beam lithography with a RAITH EBL.

The Al_2_O_3_ thin films were acquired via AP-CVD using trimethylaluminum (TMA) and H_2_O precursors. In the AP-CVD process, we bubbled the TMA liquid precursor with N_2_ gas at a flow rate of 20 sccm to generate the vapors of TMA. The TMA vapors were then mixed with 130 sccm of N_2_ gas, which works as a carrier gas to assist in TMA delivery to the reactor head. Similarly, H_2_O vapors were generated by bubbling the water with 45 sccm of N_2_ gas and were mixed with N_2_ carrier gas of 255 sccm. A separate N_2_ gas line with a flow rate of 1200 sccm was used to partially separate the precursors at the reactor surface and also to remove excess unreacted precursors or reaction by-products. The thickness of the film was controlled by adjusting the speed and number of oscillations. We set the substrate temperature and oscillation speed at 150 °C and 30 mm s^*−*1^, respectively. We grew a 6 nm thin film with nine oscillations, as verified by ellipsometry measurements. The nine oscillations of the substrate corresponded to 36 sequential exposures of both TMA and H_2_O precursors, resulting in a growth rate of 0.17 nm/cycle, which was higher than the 0.11 nm cycle^−1^ rate achieved for PEALD of Al_2_O_3_ in this work.

Devices with PEALD films were fabricated using similar steps. TMA and O_2_ plasma precursors were used, and 27, 54 and 81 cycles were used to deposit a 3, 6, and 9 nm thick film. The film was deposited at the same temperature of 150 °C using an Oxford-Cluster system with a base pressure below 10^*−*6^ torr and the load lock pumped below 10^*−*5^ torr. The vacuum chamber was first purged with Ar for 3 min to stabilize the chamber pressure and temperature. TMA bubbled with argon was used to dose the substrate for 20 ms at a pressure of 15 mtorr, followed by purging the precursor line with Ar for 1 s at a pressure of 15 mtorr. Afterward, an O_2_ flow was stabilized for 500 ms before generating plasma using radio frequency (RF) with a power of 300 W at a pressure of 15 mtorr. The substrate was exposed to the O_2_ plasma for 2 s and then purged with Ar for 1 s at a pressure of 15 mtorr.

A 100 nm thick top Aluminum (Al) electrode was deposited on insulator thin films. Angstrom E-beam evaporation system used for the deposition. The base pressure was set to 4 × 10^*−*6^ torr and the deposition rate was 3 A˚ s^*−*1^. The Al electrodes were patterned using E-Beam lithography with a RAITH EBL. Wet etching was used to remove any oxidant on top of the electrode layers.

### Electrical and materials characterization

The I–V properties of the MIM diodes were measured using a Keithley 4200-SCS semiconductor characterization system connected to a Everbeing Probe Station C-8. The thickness and refractive indices of the 3, 6, 9 nm thick films were measured using a Woollam M-2000 DI ellipsometer with a wavelength range of 200–1800 nm, utilizing the CodyLor formula model. Bruker Icon Atomic Force Microscope was utilized to characterize film uniformity and surface roughness. The chemical states of the films were determined through X-ray photoelectron spectroscopy (XPS) using a Thermo ESCALAB 25 instrument with an Al-Ka X-ray source. XPS analyses were conducted by applying the Shirley baseline and employing Gaussian fitting.

### Supplementary Information

Supplementary Information.

## Data Availability

The datasets used and/or analysed during the current study available from the corresponding author on reasonable request.
