# Supplementary material for: Manufacturing of quantum-tunneling MIM nanodiodes via rapid atmospheric CVD in terahertz band
Source: Sci Rep. 2023 Nov 25;13:20733. doi: 10.1038/s41598-023-47775-5 (PMC10676383; doi:10.1038/s41598-023-47775-5)
Supplement: Supplementary file 1 — Supplementary Information. [file 41598_2023_47775_MOESM1_ESM.pdf]

SUPPLEMENTARY MATERIAL

In the context of the Poole–Frenkel effect, the current density, J, is anticipated to show a specific correlation with the electric field, E, as described in

$$J_{PF} = E \exp\left(-\frac{q\left(\phi_T - \sqrt{\frac{qE}{\pi\varepsilon_r\varepsilon_0}}\right)}{kT}\right) \tag{3}$$

where q is the elementary charge, ϕ_T is the trap energy of the insulator, ε_r is the optical dielectric constant, ε_0 is the permittivity in free space, k is Boltzmann's constant, and T is temperature.

The formula for Schottky emission is described as:

$$J_{SE} = A^* T^2 \exp\left(-\frac{q\left(\phi_B - \sqrt{\frac{qE}{4\pi\varepsilon_r\varepsilon_0}}\right)}{kT}\right) \tag{4}$$

$$\text{where } A^* = \frac{4\pi k^2 m^*}{h^3} = \frac{120 m^*}{m_0} \tag{5}$$

A^* is the effective Richardson constant, m^* is the effective electron mass in the insulator, m_0 is the free electron mass, and $q\phi_B$ is the Schottky barrier height.

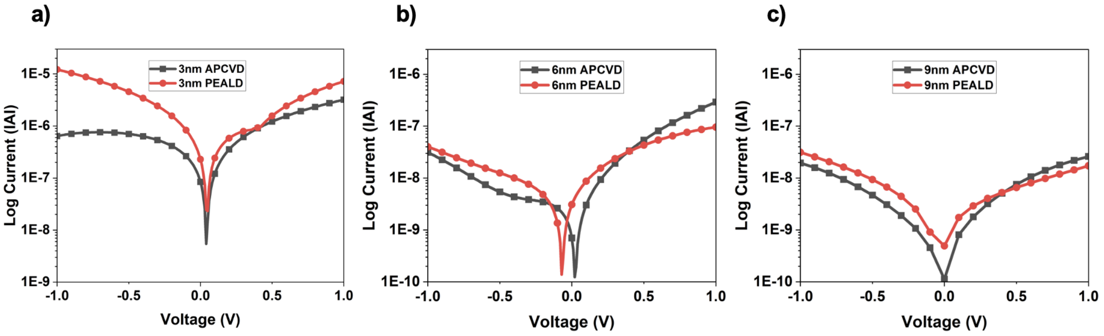

Supplementary Figure S1. I-V Curves of AP-CVD and PEALD diodes with 3nm, 6nm, and 9nm thicknesses.

Supplementary Table S1. The film compositions of Al_2O_3 films deposited using the PEALD and APCVD techniques were analyzed through X-ray Photoelectron Spectroscopy (XPS).

Technique	Film Compositions (at%)			
	O	Al	C	O/Al
PEALD	53.6	39.1	7.3	1.37
AP-CVD	55.2	36.7	8.1	1.5

Carbon was detected in both the AP-CVD and PEALD Al_2O_3 films. The presence of carbon contamination is typically attributed to the use of organometallic precursors and airborne contamination.

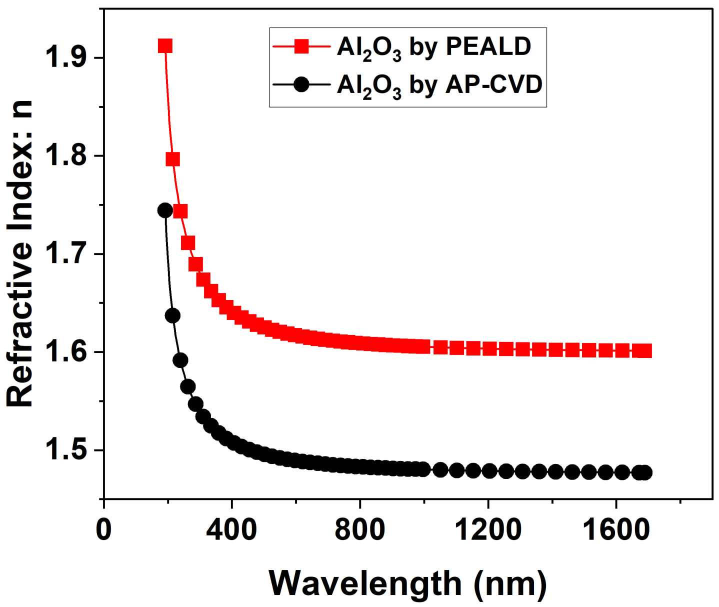

Supplementary Figure S2. Refractive indices of Al_2O_3 measured by ellipsometry for AP-CVD and PEALD films
